# Supplementary material for: DeepImmuno: Deep learning-empowered prediction and generation of immunogenic peptides for T cell immunity
Source: bioRxiv. 2020 Dec 24:2020.12.24.424262. Preprint. [Version 1] doi: 10.1101/2020.12.24.424262 (PMC7781330; doi:10.1101/2020.12.24.424262)
Supplement: 1 — Supplementary Figure 1. Workflow to generate HLA paratope sequences. To predict the antigen-binding residues of each HLA allele or paratope, we constructed consensus paratope sequences for each known human HLA allele. Shown here are five example sequences from five independent solved crystal structures of the HLA allele HLA-A*0101. A consensus paratope sequence was determined by computing the most frequent residue in each position. Another round of multiple sequence alignment generates fixed-length HLA paratopes for all HLA alleles. The token “-” is introduced to represent nicks and gaps. Supplementary Figure 2. Schematic overview of the ResNet and Graph Neural Network (GNN) models. (A) The ResNet architecture is depicted with three Residual blocks, chained together to extract high-level abstract features associated with immunogenic and non-immunogenic sequences. Each residual block encompasses three convolutional neural network layers followed by a maxpool layer. (B) The GNN architecture is depicted with the first two layers of the graph kernel designed to aggregate neighbors’ attributes (physicochemical properties of each amino acid). A mean pooling layer is used to integrate the graph-level embedding, followed by two dense fully-connected layers to predict immunogenicity. Supplementary Figure 3. Immunogenicity prediction following an ablation test of different encoding strategies across 7 evaluated algorithms. Relative immunogenicity detection performance of seven evaluated algorithms (ElasticNet, KNN, SVM, Random Forest, AdaBoost, CNN and ResNet) and three different encoding strategies (AAindex+Paratopes, One-hot encoding + Parartopes and AAindex + HLA Pseudo34). AAindex encoding and HLA paratopes representation is shown in black, Onehot encoding and HLA paratopes representation is shown in red, AAindex encoding and HLA pseudo34 sequences representation is shown in orange. The x-axis represents nine different performance evaluation statistics across the four tes [file NIHPP2020.12.24.424262-supplement-1.pdf]

Supplementary Figure 1

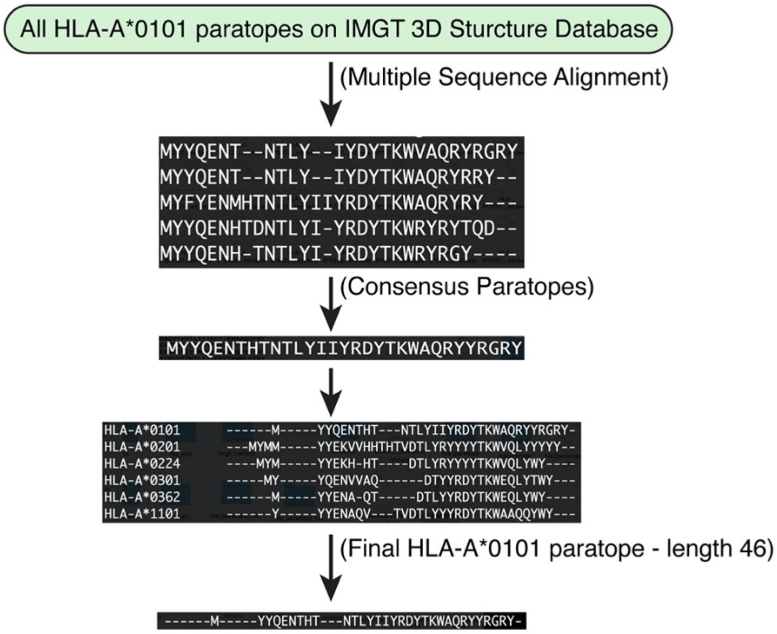

Supplementary Figure 1. Workflow to generate HLA paratope sequences. To predict the antigen-binding residues of each HLA allele or paratope, we constructed consensus paratope sequences for each known human HLA allele. Shown here are five example sequences from five independent solved crystal structures of the HLA allele HLA-A*0101. A consensus paratope sequence was determined by computing the most frequent residue in each position. Another round of multiple sequence alignment generates fixed-length HLA paratopes for all HLA alleles. The token “-” is introduced to represent nicks and gaps.

Supplementary Figure 2

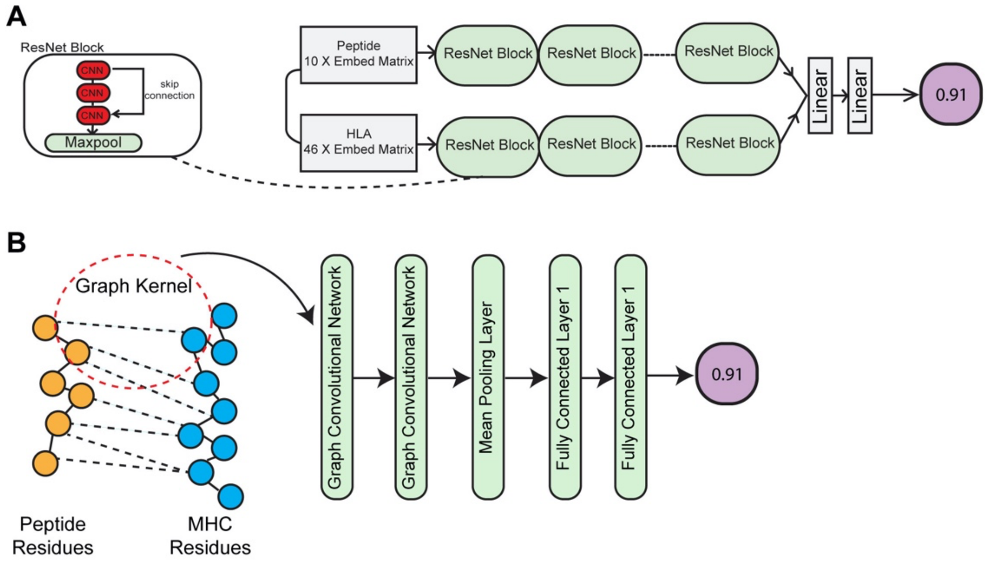

Supplementary Figure 2. Schematic overview of the ResNet and Graph Neural Network (GNN) models. (A) The ResNet architecture is depicted with three Residual blocks, chained together to extract high-level abstract features associated with immunogenic and non-immunogenic sequences. Each residual block encompasses three convolutional neural network layers followed by a maxpool layer. (B) The GNN architecture is depicted with the first two layers of the graph kernel designed to aggregate neighbors' attributes (physicochemical properties of each amino acid). A mean pooling layer is used to integrate the graph-level embedding, followed by two dense fully-connected layers to predict immunogenicity.

Supplementary Figure 3

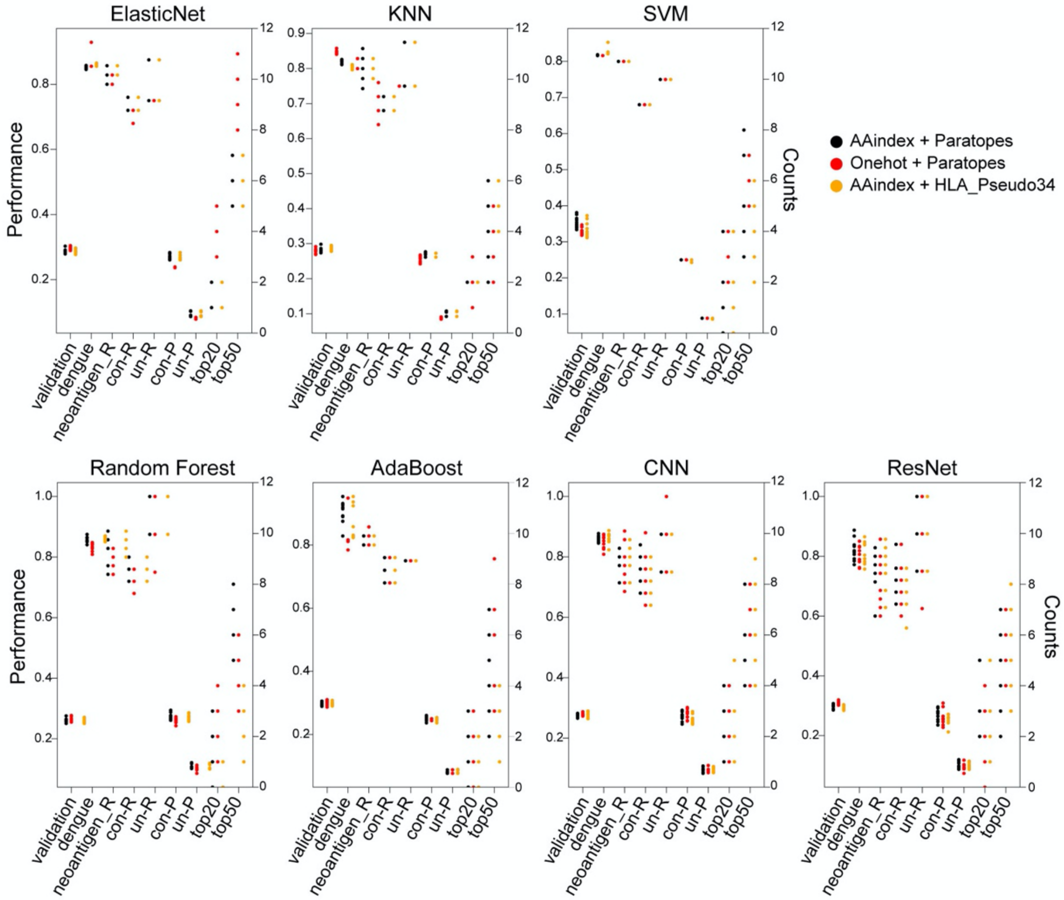

Supplementary Figure 3. Immunogenicity prediction following an ablation test of different encoding strategies across 7 evaluated algorithms. Relative immunogenicity detection performance of seven evaluated algorithms (ElasticNet, KNN, SVM, Random Forest, AdaBoost, CNN and ResNet) and three different encoding strategies (AAindex+Paratopes, One-hot encoding + Parartopes and AAindex + HLA Pseudo34). AAindex encoding and HLA paratopes representation is shown in black, Onehot encoding and HLA paratopes representation is shown in red, AAindex encoding and HLA pseudo34 sequences representation is shown in orange. The x-axis represents nine different performance evaluation statistics across the four test datasets. These statistical metrics are: 1) validation (RMSE in nested 10-fold validation dataset), 2) dengue (Accuracy in dengue virus dataset), 3) neoantigen_R (Recall in cancer neoantigen dataset) and 4) con-R (Recall in COVID-19 convalescent patients group), un-R (Recall in COVID-19 unexposed patients group), con-P (Precision in COVID-19 convalescent patients group) and un-P (Precision in COVID-19 unexposed patients group). top 20 = immunogenic neoantigen in top 20 ranked hits; top 50 = immunogenic neoantigen in top 50 ranked hits. The indicated dataset-specific performance metric is indicated on the y-axis (range 0-1, left) or top 20/50 hits (counts, right).

Supplementary Figure 4

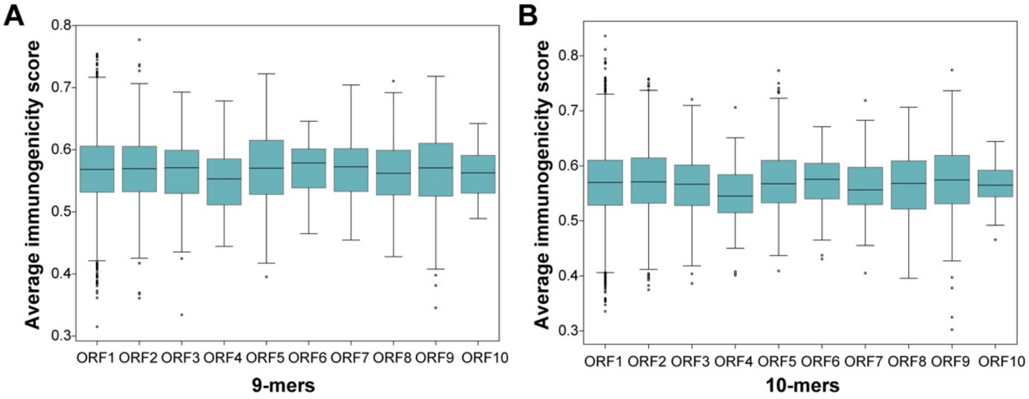

Supplementary Figure 4. Predicted immunogenicity is consistent across all ORFs in the SARS-Cov-2 proteome. (A) Predicted immunogenicity of all 9-mer peptides translated from 10 different ORFs. (B) Predicted immunogenicity of all 10-mer peptides translated from 10 different ORFs.

Supplementary Figure 5

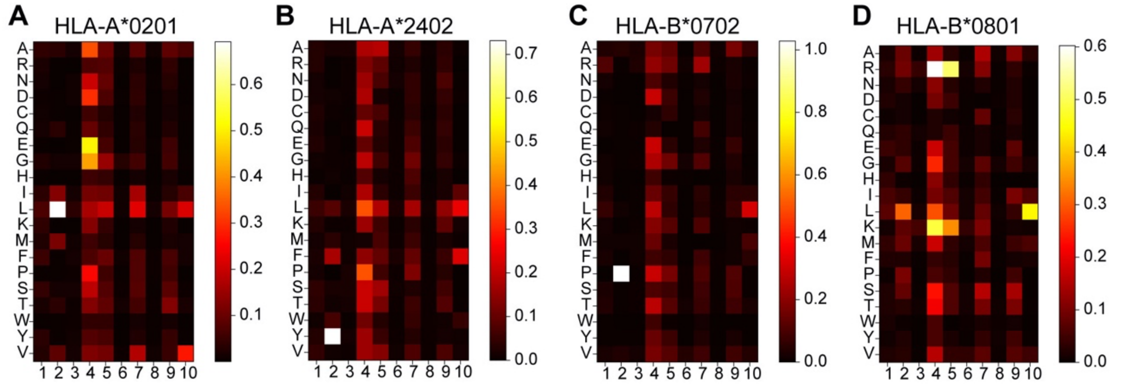

Supplementary Figure 5. Immunogenicity motif analysis reveals critical HLA-peptide-TCR interacting sequences. The immunogenic motif heatmap for (A) 2,046 HLA-A*0201, (B) 210 HLA-A*2402, (C) 162 HLA-B*0702 and (D) 176 HLA-B*0801 bound epitopes in the IEDB training dataset. Positions are weighted by their relative importance derived from the occlusion sensitivity analysis in Figure 3.

Supplementary Figure 6

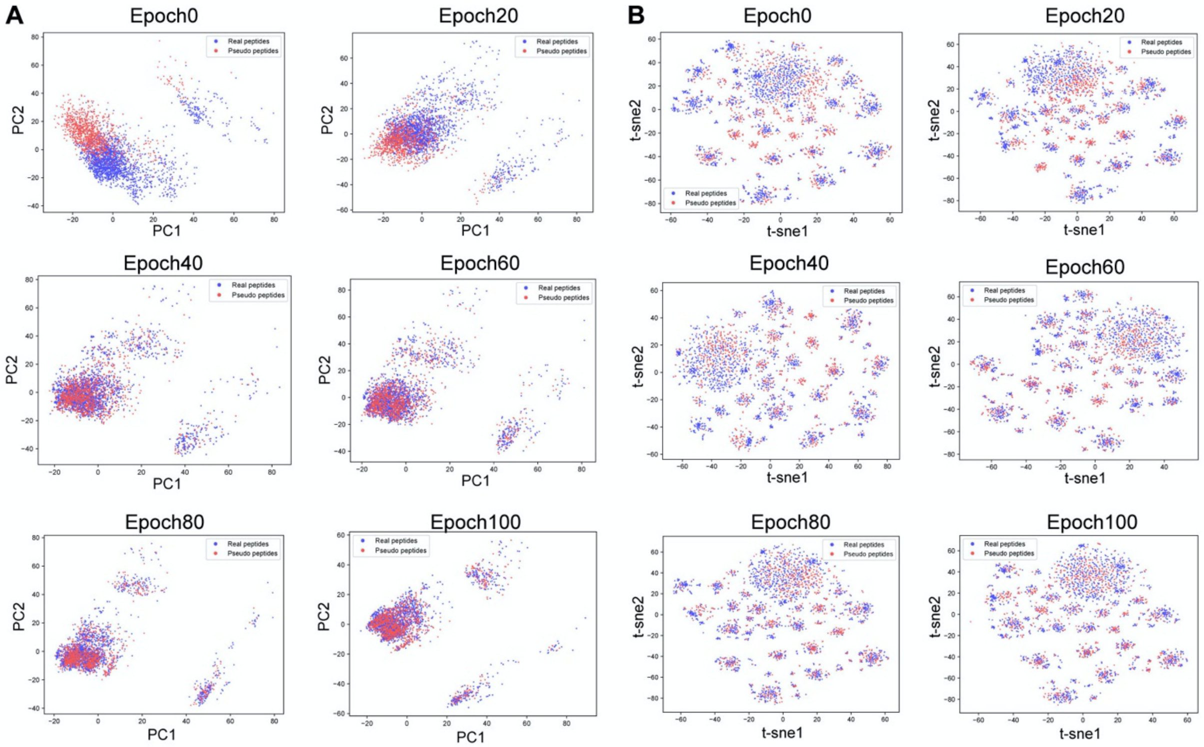

Supplementary Figure 6. Convergence of real immunogenic and pseudo-sequences with progressive GAN training. GAN generative sequences from epoch 0, epoch 20, epoch 40, epoch 60, epoch 100 were concatenated with real HLA-A*0201 instances and their joint embedding spaces were visualized using either (A) PCA or (B) t-SNE.

Supplementary Figure 7

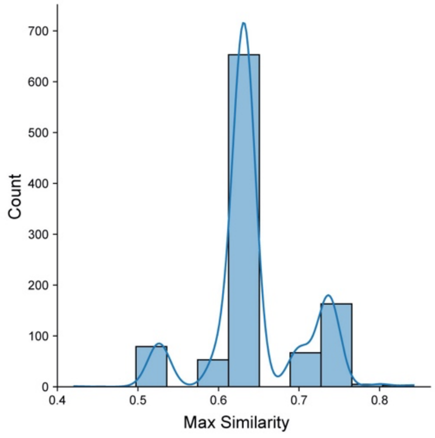

Supplementary Figure 7. Distribution of max similarity between GAN generated pseudo-sequences and their matched real immunogenic peptides in HLA-A*0201.
The maximum similarity for each GAN-generated pseudo-sequence and its most similar counterpart in real immunogenic peptide repertoires are shown, with similarity defined as the
